# Transgenic mice with mammary gland targeted expression of human cortactin do not develop (pre-malignant) breast tumors: studies in MMTV-cortactin and MMTV-cortactin/-cyclin D1 bitransgenic mice

**DOI:** 10.1186/1471-2407-6-58

**Published:** 2006-03-14

**Authors:** Agnes GSH van Rossum, Maaike PA van Bragt, Ellen Schuuring-Scholtes, Jan CM van der Ploeg, Johan HJM van Krieken, Philip M Kluin, Ed Schuuring

**Affiliations:** 1 Department of Pathology, Leiden University Medical Center, Albinusdreef 2, 2300 RC Leiden, The Netherlands; 2 Division of Cellular Biochemistry, The Netherlands Cancer Institute, Plesmanlaan 121, 1066 CX, Amsterdam, The Netherlands; 3 Department of Pathology, Radboud University Nijmegen Medical Centre, Geert Grooteplein-Zuid 10, 6500 HB, Nijmegen, The Netherlands; 4 Department of Pathology, University Medical Center Groningen, Hanzeplein 1, 9700 RB, Groningen, The Netherlands

## Abstract

**Background:**

In human breast cancers, amplification of chromosome 11q13 correlates with lymph node metastasis and increased mortality. To date, two genes located within this amplicon, *CCND1 *and *EMS1*, were considered to act as oncogenes, because overexpression of both proteins, respectively cyclin D1 and cortactin, correlated well with 11q13 amplification. Cyclin D1 is involved in cell cycle regulation and the F-actin-binding protein cortactin in cytoskeletal dynamics and cell migration. To study the role of cortactin in mammary gland tumorigenesis, we examined mouse mammary tumor virus (MMTV)-cortactin transgenic mice and MMTV-cortactin/-MMTV-cyclin D1 bitransgenic mice.

**Methods:**

MMTV-cortactin transgenic mice were generated and intercrossed with previously described MMTV-cyclin D1 transgenic mice. Immunohistochemical, Northern and Western blot analyses were performed to study the expression of human transgene cortactin during mammary gland development and in mammary tumors. For tumor incidence studies, forced-bred, multiparous mice were used to enhance transgene expression in the mammary gland. Microscopical examination was performed using haematoxylin and eosin staining.

**Results:**

Mammary gland tumors arose stochastically (incidence 21%) with a mean age of onset at 100 weeks. This incidence, however, did not exceed that of aged-matched control FVB/N mice (38%), which unexpectedly, also developed spontaneous mammary gland tumors. We mimicked 11q13 amplification by generating MMTV-cortactin/-MMTV-cyclin D1 bitransgenic mice but did not observe any synergistic effect of cortactin on cyclin D1-induced mammary hyperplasias or carcinomas, nor development of distant metastasis.

**Conclusion:**

From this study, we conclude that development of (pre-malignant) breast tumors in either wild type or MMTV-cyclin D1 mice was not augmented due to mammary gland targeted overexpression of human cortactin.

## Background

Breast cancer is the most common form of cancer among women in Western countries, with approximately one out of nine being affected in their lifetime [1]. The major cause of breast cancer mortality is the development of distant metastasis. Amplification of chromosome 11q13 is frequently detected in human carcinomas of the breast [2,3] and correlates with the presence of lymph node metastases and increased mortality [2,4,5]. Two genes located within this amplicon, *EMS1*, encoding cortactin, and *CCND1 *encoding cyclin D1, were both considered to act as oncogenes in breast cancer, because increased expression of both cortactin and cyclin D1 correlated well with 11q13 amplification [5-7].

Several lines of evidence point to an important role for both cyclin D1 as well as *EMS1*/cortactin in breast cancer formation. Cyclin D1, involved in cell cycle regulation, is a well-established human oncogene [8-10]. While the *CCND1 *gene is amplified in up to 20% of human breast cancers, cyclin D1 protein is overexpressed in over 50% of human mammary carcinomas [3,11-13]. Overexpression of cyclin D1 seems to have a causative role in breast cancer formation, since mice containing a mammary-gland targeted MMTV-cyclin D1 transgene develop mammary tumors at higher incidence (5 out of 9 transgenic mice compared to none of 15 non-transgenic controls) [14]. However, tumors appeared after a relatively long latency period of 17.5 months, suggesting that cyclin D1 is a relatively weak oncogene compared to c-neu, Ha-ras and c-myc oncogenes in MMTV-neu [15], MMTV-ras [16] or MMTV-myc transgenic mice [17] with a latency period of respectively 3, 6 and 11 months. Cyclin D1 appears to be critical in some pathways of mammary tumorigenesis, because cyclin D1-deficient mice are resistant to breast cancers induced by the c-neu and Ha-ras oncogene, but remain fully sensitive to breast cancers induced by c-myc and Wnt-1 [18].

Cortactin, identified as a prominent Src substrate, is an F-actin binding protein involved in Arp2/3-mediated actin polymerization and consequently able to modulate the F-actin cytoskeleton [19,20] and cell shape changes (reviewed by [21]). Cells overexpressing cortactin show enhanced migration [22-24], invasion [22] and increased metastatic potential *in vivo *[25]. Thus, increased expression of cortactin might promote tumor cell invasion and metastasis. Cancer development and progression is a multi-step process [26-28], therefore, since both cyclin D1 and cortactin are overexpressed in most breast carcinomas with 11q13 amplification, their cooperative action might contribute to breast cancer development.

In the present study, we generated MMTV-cortactin transgenic mice to study the effect of mammary gland targeted cortactin overexpression on morphogenesis of normal mammary gland development and the potential to induce mammary gland tumors. To mimic the 11q13 amplification and to investigate the possible cooperative action of cyclin D1 and cortactin, we generated bitransgenic cyclin D1/-cortactin mice by crossing MMTV-cyclin D1 [14] with MMTV-cortactin mice.

## Methods

### Mice and tissue preparation

**Figure 1:**
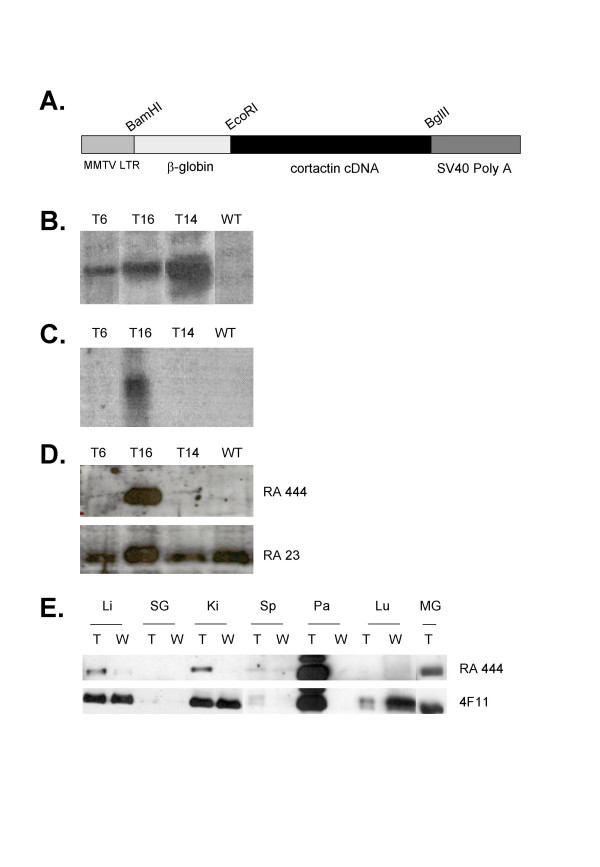
**Analysis of cortactin transgene expression**. **(A) **Structure of the MMTV-cortactin transgene construct. Vertical striped bar, 510 bp promoter/enhancer of the MMTV-LTR (-360/+150); stippled bar, 920 bp β-globin intron 2 sequence inserted to generate a stable transgene mRNA; black bar, 1.8 kb human EMS1 cDNA [29]; hatched bar, transcriptional processing sequences derived from SV40. Relevant restriction sites are indicated. **(B) **Southern blot analysis of tail DNA of three cortactin transgenic founders and one wild type (WT) mouse. The intensity of the bands corresponds to the increased copy number of the integrated transgenic cortactin cDNA. T6 contains one cDNA copy. **(C) **Northern blot analysis of 10-days lactating mammary glands. Expression of human transgenic cortactin mRNA (3,6 kb) was detected in T16 only. Full-length human cortactin cDNA was used as a probe. **(D) **Western blot analysis of 10-days lactating mammary glands to detect transgenic cortactin protein expression. Polyclonal antibody RA444 was used to recognize specific human transgene expression, whereas polyclonal antibody RA23 recognized both human and endogenous mouse cortactin. **(E) **Virgin T16 transgenic (T) and non-transgenic (W) female mice were treated with glucocorticosteroid dexamethasone to stimulate transgene expression from the MMTV promoter. Lysates from liver (Li), salivary gland (SG), kidney (Ki), spleen (Sp), pancreas (Pa), lung (Lu) and 10-days lactating mammary gland as a positive control (MG) were subjected to Western blotting. Polyclonal antibody RA444 was used to detect specific human transgene expression, whereas monoclonal 4F11 antibody recognizes both human and mouse cortactin. Transgene expression was significantly induced in the pancreas, liver and kidney.

To generate transgenic mice with targeted expression of cortactin in the mammary gland, the 1.8 kb EcoRI/BamHI fragment carrying the open reading frame of the human EMS1/cortactin cDNA (same fragment as in clone pGEM42.8/4203 [29]) was cloned into the EcoRI/BglII sites of the pJ5Ω/HG-vector (pJ5Ω/HG/EMS1/4230; see Figure 1A) [30]. The pJ5Ω/HG-vector was constructed by inserting a genomic 920 bp BamHI/EcoRI fragment including intron 2 with splice donor and acceptor sites of the human β-globin gene (provided by F. Grosveld, MRC, London) into the BamHI/EcoRI sites in pJ5Ω supplemented with a 5'-MMTV-promotor and 3'-SV40-polyadenylation site [31]. The MMTV-LTR in pJ5Ω is an authentic viral LTR obtained from the C3H mouse mammary tumor virus (MMTV) and contains the elements required for glucocorticoid regulation, promoter action and capping (-360/+150). MMTV-cortactin transgenic mice were generated in the animal colony of the Netherlands Cancer Institute (Amsterdam) under specific pathogen-free conditions by microinjection of the transgene into pro-nuclei of fertilized zygotes as described previously [32]. MMTV-cyclin D1 transgenic mice (MP1 line) were obtained from Dr. Andrew Arnold (Department of Medicine, Massachusetts General Hospital, Boston, USA.) and described previously [14]. The MMTV-cyclin D1 mice used in our experiments have retained inducible expression of cyclin D1 as detected with Northern blot analysis on mammary glands of day 10 of lactation (data not shown). Both cortactin and cyclin D1 transgenic mice were generated in the same inbred FVB/N genetic background. All mice were cross-bred and maintained under restricted conditions in the central animal house at the department of Pathology (Leiden University Medical Center). Animal care and experimentation were in accordance with legislation on animal experiments as determined by the Dutch Veterinary Inspection.

Exclusively female animals were used in the experiments, and housed with male breeders only for fertilization. Pregnancy samples were obtained on day 11 or day 17 after vaginal plugs were identified. Lactating mammary glands were obtained from mice 4, 10 or 17 days after litters were born. Involution was studied by removing pups from their mother on day 17 of lactation and obtaining samples on day 4 or 8 after weaning. For mammary gland tumor incidence experiments, mice were kept under forced-breeding conditions to continuously stimulate MMTV-driven-mammary-gland targeted transgene expression. Multiparous females underwent at least 2 but in general 3 to 7 successful pregnancies each followed by three weeks weaning. Mice showing abnormal clinical signs or discernable tumor development were sacrificed and tissues were macroscopically analyzed and prepared for further analysis. For detection of hyperplasias one abdominal mammary gland was harvested for whole-mount, the other abdominal mammary gland was frozen in isopentane (without elimination of the inguinal lymph nodes) and stored at -80°C; the second and thoracic mammary glands were fixed in 4% formaldehyde in PBS and used for histological analysis. The pathology of mammary tumors and premalignant lesions was assessed by two persons (AGSHR and JHJMK), according to the consensus report from the Annapolis meeting on the mammary pathology of genetically engineered mice [33]. Representative parts of other tissues and organs were frozen and stored at -80°C or fixed in PBS-buffered formaldehyde and prepared for histological analysis. To validate the hormonal inducibility of the MMTV-driven transgene *in vivo*, mice were injected intra-peritoneally with 0,25 ml (4 mg/ml) dexamethasone per mouse during three successive days and sacrificed on day 4.

### DNA analysis and Southern blotting

Genomic DNA was isolated from the tail tip as reported previously [32] and transgenic progeny was identified by probing equal amounts of HindIII-digested DNA, electrophoresed in a 1% agarose gel and transferred to nitrocellulose membrane (Amersham Biosciences Buckinghamshire, England). The presence of the transgene was detected by Southern blot analysis with the 1.8 kb EMS1 cDNA probe (pGEM42.8/4203 [30]) and the 0.9-kb cyclin D1 cDNA probe (p3128-CCND1, encompassing the coding domain [34]) radiolabeled with [α-^32^P] αdCTP (Amersham). The filters were washed two times at 60°C in 2× SSC; 0.1% SDS for 30 min and the presence of DNA transgene was determined by phospho-imaging analysis.

### RNA extraction and Northern blotting

Total RNA was isolated from transgenic and non-transgenic frozen tissues using the TRIZOL reagent (GIBCO Invitrogen, Carlsbad, CA, USA) according to the manufacturer's protocol. Northern blotting was performed as previously described [34]. In brief, 10 μg RNA was size fractionated on a 1% agarose 1% formaldehyde gel, transferred with 20× SSC (3 M NaCl and 0.3 M sodium citrate) onto a nitrocellulose membrane (Amersham), washed in 3× SSC, dried, and baked at 80°C for 4 hours. Blots were hybridized with the [^32^P]-αdCTP-labeled 1.8-kb EMS1 cDNA probe (pGEM42.8/4203 [30]) at 55°C in sodium phosphate hybridization mix (0,5 M Na_2_HPO_4_, 0,5 M NaH_2_PO_4_, 7% SDS, 1 mM EDTA, 1% BSA, pH = 7). The filters were washed two times at 60°C in 0,1× SSC;1% SDS for 15 min and RNA transgene expression was determined by phospho-imaging analysis.

### Protein extraction and Western blotting

Approximately 10 frozen sections (20 μm) of frozen transgenic and non-transgenic tissue were dissolved in Hot-SDS (1%SDS/10 mM EDTA and 1 tablet of complete protease inhibitors (Roche, Mannheim, Germany) per 50 ml lysisbuffer). The protein concentration was determined using the Biorad Protein assay kit. Lysates (100 μg protein) added with Laemmli sample buffer were boiled and separated by 10% SDS-PAGE. Proteins were blotted onto nitrocellulose and filters were blocked in Tris-Buffered Saline Tween-20 (TBST) containing 5% non-fat dried milk for at least 1 h. To specifically detect transgenic human cortactin, Western blots were incubated with the polyclonal antibody RA444 [30] or monoclonal anti-cortactin Esab157 (P13320-clone 30 from BD Biosciences Pharmingen, USA). Both these antibodies did not cross-react with mouse cortactin ([30] and data not shown). Blots were stripped and reincubated with monoclonal 4F11 or polyclonal antibody RA23 to detect both transgene (human) and endogenous (mouse) cortactin [30]. A monoclonal antibody against actin (Mab1501R, Chemicon, Temecula, CA, USA) was used as loading control. Blots were incubated with secondary anti-mouse or anti-rabbit IgG-peroxidase conjugate (BD Biosciences Pharmingen) and developed using the ECL enhanced chemiluminescent detection system (Amersham).

### Histology and immunohistochemical staining

From each sacrificed mouse, various tissues (lung, brain, sketal muscle, heart, femur, spleen, kidney, mammary glands [see first paragraph], salivary glands, colon, stomach, uterus/ovary, liver, pancreas, lymph nodes) were fixed in 4% formaldehyde in PBS, embedded in paraffin, sectioned at 4 μm, routinely stained with haematoxylin and eosin (HE) and microscopically examined by a pathologist. For immunohistochemical staining of the paraffin-embedded sections antigen retrieval was performed by boiling the sections in a 0,01 M sodium citrate buffer at pH = 6 for 10 min. Then, sections were blocked for 30 min with 0,5% casein in PBS. The monoclonal antibody against cortactin (P13320) was conjugated with FITC (SPC2476, BD Biosciences Pharmingen, USA) and used as primary antibody in a dilution of 1:900 in PBS/1% BSA and incubated overnight at 4°C. This antibody was used because conventional mouse monoclonal antibodies lead to background staining on mouse sections when used in combination with a secondary rabbit anti-mouse antibody. As secondary antibody, sheep anti-fluorescein-POD (peroxidase conjugated) Fab fragments (Roche) was used in a 1:300 dilution at room temperature for 30 min, followed by treatment with DAB substrate plus H_2_O_2 _(0.33%) (Merck, Darmstadt, Germany) for 10 min. Sections were lightly stained with haematoxylin and embedded in mounting medium. The pancreas from each mouse was as a positive control for staining.

### Whole-mount staining

Abdominal mammary fat pads used for whole mount staining were dissected from the pelt of transgenic and control mice, spread on plastic gauze and fixed in 1:3 acetic acid/ethanol solution for 60 min. The mammary fat pads were than dehydrated in 70% ethanol (15 min), rinsed in demineralised water, and stained overnight with alum carmine (1 g carmine, 2.5 g alum potassium sulphate in 500 ml Millipore water). The stained pads were washed in a series of ethanol steps (70%, 95%, and 100%, respectively), and transferred to xylene for 1 hr. The stained whole mounts were stored in methyl salicylate. The presence of MIN or HAN was scored by eye and by analysis of pictures of the whole mounts.

### Statistical analysis

Survival curves of mice were analyzed with the statistical software of SPSS 10, which uses log-rank tests to determine statistically significant differences between (tumor-free) survival curves.

## Results

### Generation of MMTV-cortactin transgenic mice

To obtain transgenic mice that overexpress the human cortactin protein in mammary epithelium, a construct was generated using the 1.8-kb cDNA sequence encoding human cortactin under transcription control of the steroid-inducible mouse mammary tumor virus (MMTV)-promoter (Fig. 1A). To test the steroid-inducibility of the transgene, the MMTV-cortactin construct was transfected in CAC-L153 mouse fibroblasts. Upon addition of the glucocorticoid dexamethasone, the expression of the human transgene was induced 10–40 fold compared to unstimulated transfectants as reported previously [30] (data not shown). Transgenic mice were generated by microinjecting fertilized oocytes from FVB/N inbred mice with this MMTV-cortactin construct. Three independent MMTV-cortactin founders were selected with a low (T6), an intermediate (T16) and a high (T14) DNA copy number of the transgene (Fig. 1B). To determine transgene expression, total mRNA and protein were isolated from the abdominal mammary gland (MG) after 10-days of lactation. In transgenic line T16, the human cortactin transgene was highly expressed at both mRNA (Fig. 1C) and protein level (Fig. 1D), whereas the transgene was undetectable in lines T6 and T14. Also at other stages of mammary gland development, no transgene expression was detected in T6 and T14 (data not shown). The T16 human cortactin transgenic mice were selected for further analysis.

We examined the expression pattern of the T16 transgene in several tissues using Western blotting (Fig. 1E) and immunohistochemistry (data not shown). In virgin transgenic mice, we detected transgene cortactin expression only in the pancreas and not in other tissues including the mammary gland (MG), salivary gland, lung, liver, spleen, kidney and muscle (data not shown). When virgin mice were treated with the glucocorticosteroid dexamethasone to stimulate the MMTV promoter, transgene expression was significantly induced in the MG (data not shown), pancreas, liver, and kidney (Fig. 1E). T16 transgenic mice developed normally, were fertile and had no overt phenotypic differences from wild-type mice. In male mice, transgene expression was observed only in a few cells of the prostate, but not in the MG or any other tissues/organs (data not shown) and, therefore, no further analysis was pursued with MMTV-cortactin transgenic male mice. Thus, in the transgenic mouse line T16, expression of the MMTV-driven human cortactin transgene is targeted to the mammary gland.

### Normal mammary gland development in MMTV-cortactin transgenic mice

**Figure 2:**
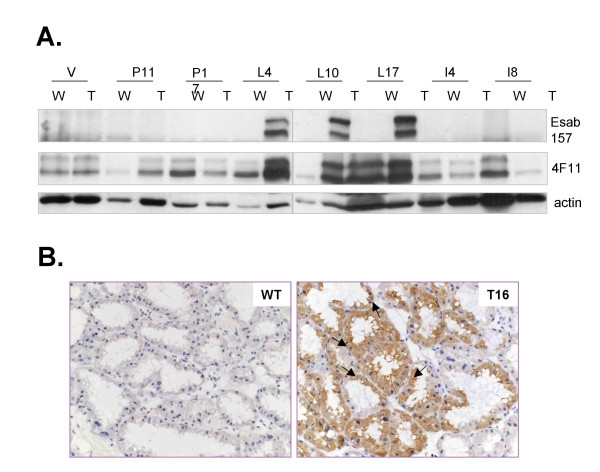
**Normal mammary gland development in cortactin transgene mice**. **(A) **Expression of endogenous and transgenic cortactin in mouse mammary epithelium at different stages of mammary gland development as determined by Western blot analysis. Total protein was loaded from the abdominal mammary gland of non-transgenic (W) and transgenic mouse line 16 (T) of virgin female mice (V), mice at day 11 and 17 of pregnancy (P11, P17), day 4, 10 and 17 of lactation (L4, L10, L17) and day 4 and 8 of involution (I4, I8). Monoclonal antibody Esab157 was used to recognize specific human transgene expression, whereas monoclonal antibody 4F11 recognizes both human and mouse cortactin. Actin was used as a loading control. **(B) **Immunohistochemical detection of cortactin transgene in a 10-days lactating mammary gland (L10). WT, wild type; T16, transgenic mouse line 16. About 50% of epithelial cells from the transgenic section stained positive for cortactin. Arrows indicate cortactin localization at cell-cell contacts. Sections were counterstained with haematoxylin.

To determine the temporal pattern of expression of the cortactin transgene throughout MG development, the abdominal MG of both T16 transgenic and non-transgenic FVB/N inbred mice were obtained from virgin (V) mice, from mice at day 11 and 17 of pregnancy (P11, P17), from day 4, 10 and 17 of lactation (L4, L10, L17) and from day 4 and 8 of involution (I4, I8). All MGs were analyzed by Northern blotting (data not shown), Western blotting (Fig. 2A) and immunohistochemistry (Fig. 2B and data not shown). The cortactin transgene was highly expressed during lactation (L4, L10, L17), but not during pregnancy and involution, whereas endogenous cortactin was expressed during all stages in both WT and transgenic mice (Fig. 2A). Immunohistochemistry (IHC) revealed that the cortactin transgene was expressed in approximately 50% of the mammary epithelial cells and localized in the cytoplasm, at the apical site of the cell (at the lumen) and in cell-cell contacts (arrows), which is in agreement with our *in vitro *data [35]. In all stages of MG development, no macroscopic (whole-mounts) or microscopic (HE and IHC stainings) morphological changes were observed in the MMTV-cortactin mice compared to the WT mice (Fig. 2B for L10, Fig. 4A for virgin, other stages data not shown). In agreement with these observations, T16 transgenic mice are able to normally breastfeed their pups. Taken together, MMTV-cortactin mice express human cortactin at high levels in the lactating MG without any detectable morphological consequences.

### MMTV-cortactin transgenic mice neither develop (pre-malignant) breast tumors nor accelerate MMTV-cyclin D1-induced mammary tumorigenesis

The role of cortactin overexpression on MG tumor development *in vivo*, either in the absence or presence of co-expression of cyclin D1, was studied in a cohort of 100 multiparous mice that underwent multiple pregnancies/lactations to force transgene expression in the MG. By intercrossing MMTV-cortactin mice (T16) with MMTV-cyclin D1 mice (MP1 [14]), the cohort was composed of 28 MMTV-cortactin mice (T16), 15 MMTV-cyclin D1 mice (MP1), 23 MMTV-cortactin/MMTV-cyclin D1 mice (T16*MP1) and 34 wild-type mice (WT, MMTV-cortactin and MMTV-cyclin D1 negative mice). MG tumors were observed in 6 out of 28 MMTV-cortactin T16 transgenic mice (21%). By histological examination 6 tumors were classified as adeno(squamous) carcinomas (Table 1 and 2). One mouse had a tumor partly consisting of adeno and partly of spindle cell carcinoma. One mouse had two tumors, an adenosquamous and a cribriform carcinoma. For breast tumorigenesis studies, we have used FVB/N strain to generate transgenic mice because of its reported low incidence of MG tumors [36]. Unexpectedly, in our cohort of 34 WT FVB/N mice, we observed 13 mice with MG carcinomas (38%, 11 adeno(squamous), 3 spindle cell, 1 cribriform and 1 endocrine carcinoid, Table 1 and 2). MG tumors were observed in 6 out of 15 (40%) MMTV-cyclin D1 mice: 4 adenosquamous, 1 papillary, 1 cribriform and 1 endocrine carcinoid carcinoma. One mouse had even 3 tumors. And finally, in the cohort of 23 bitransgenic T16*MP1 mice, 7 mice bore MG tumors (30%), 6 adeno(squamous) and 1 papillary carcinoma (Table 1 and 2). These data revealed that the incidence of MG tumors in the non-transgenic FVB/N mice (38%) is similar to MMTV-cyclin D1 (40%) mice and almost twice as high than in MMTV-cortactin (21%) or MMTV-cyclin D1/cortactin bitransgenic mice (30%). When comparing the histology of the MG tumors between the transgenic and WT mice, it is remarkable that most MG tumors from WT, T16 or T16*MP1 mice are of adeno(squamous) origin (11/16, 6/7 and 6/7, respectively). Thus, the origin of MG tumors in MMTV-cortactin mice is comparable to WT mice. In MMTV-cyclin D1 (MP1) mice only half of the tumors are from adeno(squamous) origin (4/8), which is in agreement with previous results [14]. This suggests that the origin of the non-adenosquamous carcinomas in MMTV-cyclin D1 mice might be caused by cyclin D1 expression and might be repressed in the T16*MP1 bitransgenic mice. In summary, our data suggest that the MMTV-cortactin transgenic mice neither develop breast tumors nor accelerate MMTV-cyclin D1-induced mammary tumorigenesis on top of the causal MG tumors that arose in the aged FVB/N mice.

**Figure 3:**
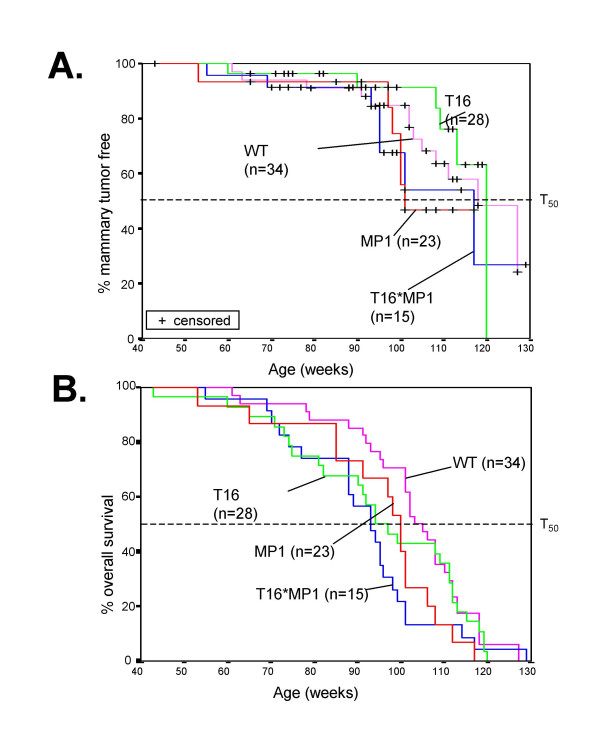
**Tumor incidence in transgenic mice**. **(A) **Kaplan-Meier analysis of the occurrence of mammary gland tumors in female wild type (WT), cortactin transgenic (T16), cyclin D1 transgenic (MP1) and cortactin/cyclin D1 bitransgenic (T16*MP1) mice as indicated by % mammary tumor free. No significant differences were seen. The age at which 50% of the animals were found to have mammary gland tumors, the T_50_, is for each mouse line more than 100 weeks. n, number of mice examined; +, indicates those mice that died without a MG tumor (censored). **(B) **Kaplan-Meier analysis determining the survival ratio of wild type and transgenic mice. MP1 and T16*MP1 were significantly different from WT (p = 0,034 and p = 0,017 respectively, log-rank test).

In figure 3A, the incidence of mammary tumors is depicted as the appearance of mammary tumor as function of time (% mammary tumor free mice). None of the three transgenic lines showed a statistically significant earlier onset of MG tumor development compared to WT FVB/N (log rank test). MG tumors of all four genotypes arose stochastically with a long latency period (mean age of MG tumor onset was between 89 and 100 weeks, Table 1). From figure 3A, we can conclude that most mice in our cohort were not sacrificed because of the appearance of MG tumors (indicated as +, censored). Therefore, we performed Kaplan Meier overall survival curves (Fig. 3B) that revealed that only MMTV-cyclin D1 (MP1) and bitransgenic (T16*MP1) (but not MMTV-cortactin transgenic) mice died significantly earlier than WT mice (p = 0,034 and p = 0,017, respectively, log rank test). Taken together, we conclude that the development of malignant breast tumors in either WT or MMTV-cyclin D1 mice was not increased due to overexpression of human cortactin.

**Figure 4:**
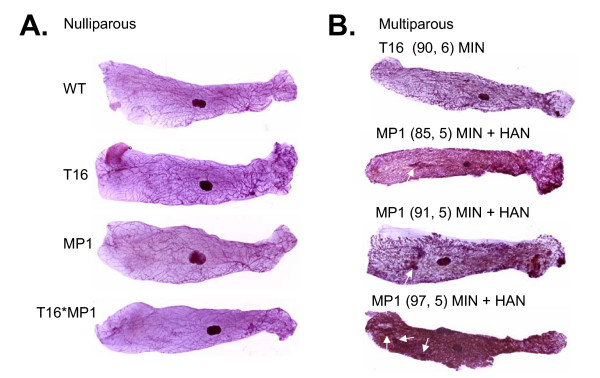
**Normal mammary epithelium in cortactin and cyclin D1 nulliparous, but not in multiparous transgenic mice**. **(A) **Whole mounts of the abdominal mammary gland from nulliparous mice of wild type (WT), cortactin transgenic (T16), cyclin D1 transgenic (MP1) and cortactin/cyclin D1 bitransgenic (T16*MP1) mice at 20 weeks of age. The dark spot in the middle represents a lymph node. **(B) **Whole mounts of the abdominal mammary gland of post-weaned multiparous mice with mammary intraepithelial neoplasia (MIN) and hyperplastic alveolar nodules (HAN, arrows). The age in weeks and the number of pregnancies that the mouse went through, are indicated between brackets.

**Figure 5:**
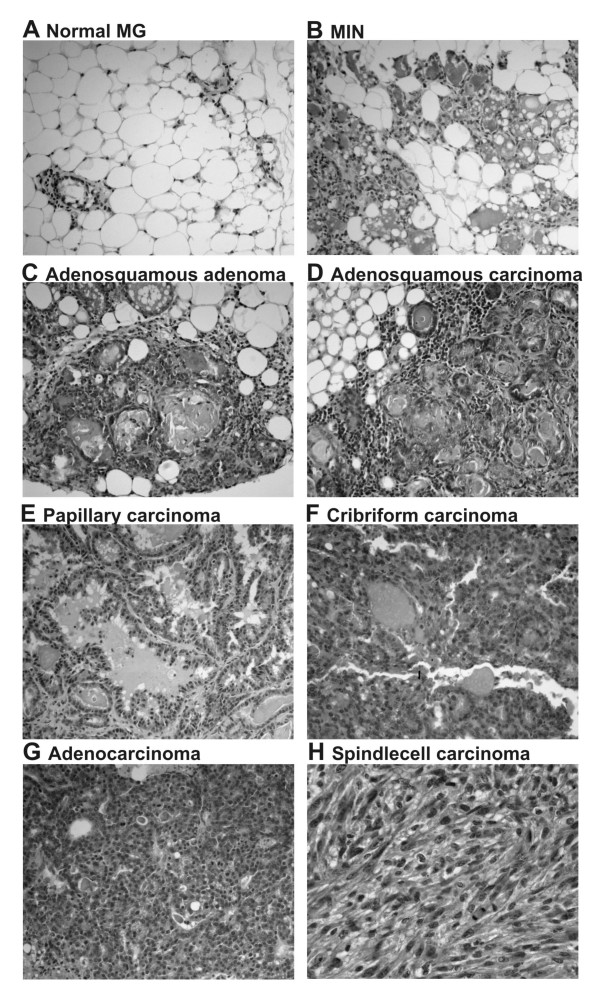
**Abnormal mammary gland morphology**. Photographs of HE stained sections of representative abdominal mammary glands of post-weaned multiparous mice with **(A) **normal mammary gland epithelium; **(B) **mammary intraepithelial neoplasia (MIN); **(C) **hyperplastic alveolar nodules (HAN) or adenosquamous adenoma; **(D) **adenosquamous carcinoma; **(E) **papillary carcinoma; **(F) **cribriform carcinoma; **(G) **adeno carcinoma; and **(H) **spindle cell carcinoma. All pictures are taken with the same magnification except (H), which is taken with a two times higher magnification.

Two month old nulliparous MMTV-cyclin D1 MP1 females have been shown to develop hyperplasia [14]. We examined the occurrence of pre-malignant lesions as determined by mammary intraepithelial neoplasia (MIN) and hyperplastic alveolar nodules (HAN) in whole mounts and HE stainings of mammary glands of 20 (Fig. 4A), 29 and 35 weeks (data not shown) old nulliparous WT, T16, MP1 and T16*MP1 mice. We did not observe hyperplastic lesion in any of the nulliparous mice of these ages. In contrast, we observed many MIN with or without HAN in older multiparous mice (Fig. 4B whole mounts and Fig. 5A,B). In fact, we observed pre-cancerous MIN and HAN lesions in all four transgenic lines (Table 1) and no significant differences were found compared to WT FVB/N mice. Immunohistochemical stainings revealed that only 1 out of 21 examined MGs of the T16 line showed transgenic cortactin expression in HAN and none of the 14 examined MGs from line T16*MP1. In addition, we did not detect any cortactin transgene expression in MG tumors of either T16 or T16*MP1 bitransgenic mice (5 MG tumors examined of each line, data not shown). Taken together, none of the MG abnormalities observed in MMTV-cortactin mice were different from WT FVB/N mice. In agreement with this, none of the carcinomas and pre-cancerous lesions showed transgene expression despite high transgene expression during lactation in this transgenic mouse strain.

More recently, from cell biological and biochemical analyses it is clear that cortactin is involved in remodeling the actin cytoskeleton via Arp2/3 mediated actin polymerization, especially its new role in mediating intercellular adhesions and cell spreading [35,37,38]. Deregulation of these processes by overexpression of cortactin might mediate the migratory and invasive potential of tumor cells. This implies that mammary gland tumors in the MMTV-cortactin transgenic mice might show an increased incidence of distant metastasis. For that purpose we screened microscopically numerous organs (especially the lung and liver) for the presence of metastatic breast tumor cells at time the mice were sacrificed. We observed lung tumors in 2 of 6 (line T16) and 3 of 7 (line T16*MP1) mice with MG tumors (Table 2). However, histological examination excluded them as being metastasis but identified them as primary tumors. Moreover, in our cohort of multiparous mice, we observed a lung-tumor incidence between 30% and 50% in all four transgenic lines (data not shown), indicating a relatively high background of spontaneous lung tumors without any direct link to MG tumors.

## Discussion

We investigated the involvement of cortactin in mammary gland tumorigenesis in MMTV-cortactin and MMTV-cortactin/-cyclin D1 bitransgenic mice. Cooperative action of cortactin in an MMTV-cyclin D1 background might affect the onset, the type or the aggressiveness of MG tumors (invasion or formation of metastasis). In this study, we found that cortactin did not provide any advantage in mammary gland tumor development. However, several circumstances might have influenced our results.

**Firstly**, the site of integration of a transgene can influence the timing of the transgene expression and may be responsible for the lack of an effect of cortactin on tumorigenesis. Indeed, the cortactin transgenic lines T6 and T14 with respectively a low and high copy number of the transgene, did not express the cortactin transgene during any stage of gestation. This indicates that the integration site and/or the copy number somehow determine the accessibility of the MMTV promoter. On the other hand, in the cortactin transgenic line T16, we observed transgene expression but that was restricted to the stage of lactation and not detectable during pregnancy, involution or in the virgin MG. For tumor development generally cell division is necessary. The fact that the cortactin transgene was not (over)expressed during pregnancy, the phase of cell division in the mammary gland, implies that the window of increased cortactin might have been too narrow to affect mammary gland tumorigenesis. This is most probably not due to the use of MMTV promoter itself, because in several other transgenic mice models to study mammary tumorigenesis such as MMTV-kgf [39] and MMTV-Cdc25B [40], the MMTV-driven transgene was expressed during all stages of gestation. Thus, transgene integration may have influenced expression pattern of the transgene in line T16 and may explain the lack of effect of cortactin on tumorigenesis.

**Secondly**, the MMTV-cortactin and MMTV-cyclin D1 mice were generated in a FVB/N inbred background. The FVB/N strain was created in the early 1970s and has since been extensively used in transgenic research because of its well-defined inbred background, superior reproductive performance, and prominent pronuclei of fertilized zygotes, which facilitates microinjection of the transgene [41]. Little is known, however, about the survival and spontaneous diseases of non-transgenic FVB/N multiparous mice. In agreement with our cohort of multiparous FVB/N mice, recently Nieto et al. [42] also observed spontaneous mammary hyperplasia and mammary tumors in 4 out of 6 multiparous mice younger than 70 weeks. In some cases mammary hyperplasia was due to pituitary abnormalities [43], because prolactin secreted by the pituitary gland, is an important stimulator of mammary gland development [44]. We observed three enlarged pituitaries in multiparous mice bearing mammary gland tumors (Table 2) indicating that these mammary gland tumors might be the consequence of a pituitary disorder. However, we did not examine prolactin concentrations to study this possibility. In transgenic mice with a strong penetrance of the transgene such as c-neu, Ha-ras or c-myc [15-17], the onset of mammary gland tumors was so early (mean age of 3, 6, and 11 months, respectively) that spontaneous diseases due to the background of the strain did not interfere with the results. However, in transgenic mice with a very low penetrance, tumors arise stochastically with a very long latency period. Furthermore, palpable mammary gland tumors, even when very large (> 1,5 cm), are never lethal by itself. This means that mice get old and finally die because of age related diseases [36]. As a result, specific phenotypes of transgenes with a low penetrance are not able to manifest themselves or were not observed due to the background disorders of aged-matched mice. In our study, overexpression of human cortactin did not accelerate the development of (pre-malignant) breast tumors (Fig. 3A) and had no significant effect on overall survival (Fig. 3B) in either WT or MMTV-cyclin D1 mice, suggesting that cortactin also might act as a low penetrance transgene.

In our cohort of 15 MMTV-cyclin D1 mice, we observed 6 mice with mammary gland tumors (40% incidence) at a mean age of 91 weeks. In the original study, 5 out of 9 MMTV-cyclin D1 (MP1) mice developed mammary tumors with a mean age of 76 weeks [8]. In another study with MMTV-cyclin D1 (MP1) transgenic mice, 8 out of 16 mice developed mammary tumors with a mean age of 113 weeks (in an FVB/N versus FVB/SV129 background) [45]. The reason for this discrepancy in the timing of mammary tumor development is unclear and is in our opinion not only due to the mixed (FVB/N/SV129) genetic background as was suggested because we also observed a long latency in a full FVB/N background. In addition, in the latter study, mammary gland tumor incidence of MMTV-cyclin D1 did not achieve statistical significance compared to wild type mice that developed MG tumors in 3 out of 10 mice as well, which is in agreement with our study (this manuscript) and the findings of Wakefield et al. [43]. Furthermore, we did not observe hyperplasia in nulliparous MMTV-cyclin D1 female mice as has previously been shown [14] nor in nulliparous MMTV-cortactin/-cyclin D1 bitransgenic mice. We did observe premalignant lesions in all multiparous transgenic mice, but also in multiparous FVB/N control mice. In addition, premalignant lesions did not express the cortactin transgene. Taken together, our cohort of multiparous MMTV-cyclin D1 mice was less potent in promoting mammary hyperplasias and carcinomas as has previously been shown and MMTV-cortactin did not accelerate the incidence of hyperplasia or MG tumors in bitransgenic mice. This is due to the long latency period and the background disorders of the FVB/N strain, which emphasizes the importance of control animals.

**Thirdly**, in human cancer overexpression of cortactin is correlated with metastasis formation. Cells overexpressing cortactin show enhanced migration and invasion *in vitro *[22-24]. In addition, overexpression of cortactin in MDA-MB-231 breast cancer cells did not affect the growth rate, but promoted the formation of bone metastases [25]. In this study, we did not observe metastases in the MMTV-cortactin (bi)transgenic mice bearing MG tumors, which might be due to the long latency of MG tumor onset and the short lifetime of the mice. It is also possible that micrometastases have developed in the MMTV-cortactin (bi)transgenic mice, but were not detected by the analysis we performed. An effect of cortactin overexpression on metastasis formation *in vivo*, can possibly be achieved by crossing the MMTV-cortactin mice with high penetrance transgenic mice (e.g. MMTV-myc, -ras or -neu) to accelerate the onset of mammary gland formation. This will give the primary tumor the opportunity to develop metastasis during a mouse lifespan.

Our results demonstrate that mammary gland targeted cortactin did not affect mammary epithelial cell morphology and did not provide proliferative advantages in the development of (pre-malignant) breast tumor in either WT or MMTV-cyclin D1 transgenic mice. In conclusion, at least in our transgenic model cortactin seems not to have a causative role in breast cancer, but appears to be a weak mammary oncogene. This study further supports the notion that cancer development and progression is a multi-step process and mutation of two or more genes may be required for cell transformation in mammary epithelial cells.

Our analysis demonstrated a high incidence of benign and malignant abnormalities in the mammary glands in the parental "control" FVB mice (see above). Many transgenic mouse models for breast cancer are generated in a background of FVB [41], however, our study (this manuscript) and two recent studies [42,43,45] illustrate that this FVB-background might not be suitable for all breast cancer studies. In addition, we observed many more abnormalities in other organs (not shown), the most common being primary lung carcinomas at an incidence of ~40% in parous as well as non-parous old FVB mice (data not shown) which is consistent with previously reported data [36]. The high incidence of these abnormalities in the mammary gland, lung and other organs has important consequences for the interpretation of new phenotypes in transgenic mice in a FVB-background.

## Conclusion

To mimic the 11q13 amplification, as seen in several human cancers, and to investigate the involvement of cortactin in mammary gland tumorigenesis and a possible cooperative action of cortactin and cyclin D1, we generated MMTV-cortactin transgenic and MMTV-cyclin D1/-cortactin bitransgenic mice. From this study, we can conclude that development of (pre-malignant) breast tumors in either wild type or MMTV-cyclin D1 mice was not augmented due to mammary gland targeted overexpression of human cortactin. In addition, our analysis demonstrated a high incidence of benign and malignant abnormalities in the mammary glands in the parental "control" FVB mice, which have important consequences for the interpretation of new phenotypes in transgenic mice in a FVB-background.

## List of abbreviations

HAN, hyperplastic alveolar nodules; HE, haematoxylin and eosin staining; IHC, immunohistochemistry; MG, mammary gland; MIN, mammary intraepithelial neoplasia; MMTV-LTR, mouse mammary tumor virus-long terminal repeat; WT, wild type.

## Competing interests

The author(s) declare that they have no competing interests.

## Authors' contributions

AGSHvR and MPAvB performed Northern and Western blot analysis. AGSHvR, MPAvB and JCMvdP took care of the mice, sacrificed them and collected tissues. ES and ESS generated the transgenic construct and performed initial screening and selection of the MMTV-cortactin transgenic mice. AGSHvR performed immunohistochemical, Southern blot and whole mount analysis, designed and drafted the manuscript. JHJMvK provided the expertise in classifying and analyzing all of the mammary tumors and participated in interpretation of study results. PMK read the manuscript and provided comments. ES applied for and received funding for this project, supervised the study and participated in writing the paper. All authors read and approved the final manuscript.

## Pre-publication history

The pre-publication history for this paper can be accessed here:



## Figures and Tables

**Table 1:** The incidence of mammary gland tumors in multiparous mice. The pathology of mammary tumors and premalignant lesions were assessed according to the consensus report from the Annapolis meeting on the mammary pathology of genetically engineered mice [33]. WT, wild type; T16, MMTV-cortactin mice; MP1, MMTV-cyclin D1 mice; T16*MP1, bitransgenic mice; wk, weeks; % MG T = no. of mice with mammary gland tumors per total no. mice; a.s.car = adeno(squamous)carcinoma of the MG; other MG car. = other MG carcinoma (see table 2); Some mice beared more than one MG carcinoma (see table 2). MIN = mammary intraepithelial neoplasia; HAN = hyperplastic alveolar nodules. MIN+HAN = no. of mice bearing MIN and/or HAN.

	**No. of mice**	**% MG T**	**a.s.car. MG**	**other MG car.**	**mean age of MG tumor onset (wk)**	MIN	HAN	HAN+MIN
**WT FVB/N**	34	13 (38%)	11 (32%)	5 (15%)	97	21 (62%)	22 (65%)	26 (76%)
**T16 (cortactin)**	28	6 (21%)	6 (21%)	1 (4%)	100	15 (55%)	19 (68%)	23 (82%)
**MP1 (cyclin D1)**	15	6 (40%)	4 (27%)	4 (27%)	91	7 (47%)	9 (60%)	13 (87%)
T16 * MP1	23	7 (30%)	6 (26%)	1 (4%)	89	8 (35%)	14 (61%)	15 (65%)

**Table 2:** Description of animals bearing mammary gland tumors. The pathology of mammary tumors and premalignant lesions were assessed according to the consensus report from the Annapolis meeting on the mammary pathology of genetically engineered mice [33]. WT, wild type; T16, MMTV-cortactin mice; MP1, MMTV-cyclin D1 mice; T16*MP1, bitransgenic mice; wk, weeks; pregn, pregnancies; MG, mammary gland; d, diameter; cm, centimeter; HAN, hyperplastic alveolar nodules; MIN, mammary intraepithelial neoplasia; hyp, hypofyse enlargement (+); carc, carcinoma; IHC-, cortactin immunohistochemical staining negative; Y, yes; N, no; nd, not done.

**Animal**	**Age (wk)**	**No. of pregn**	**MG tumor**	**Size (d,cm)**	**No. of HAN**	**MIN**	**Lung tumor**	**Hyp**	**Metas tasis**
**WT**									
**N.1**	103	4	spindle cell	0.8	7	Y	N	-	
**N.2**	93	5	adenosquamous carc. + transformation to spindle cell	1.0	3	Y	N	-	
**N.3**	108	6	2 adenosquamous carc. + 1 cribriform	1.0 0.4	8	Y	Y, carc.	+	
**N.4**	111	4	adenosquamous carc.	0.3	3	N	Y	-	
**N.5**	102	6	adeno carc.	0.8	6	Y	N	-	
**N.6**	127	5	adenosquamous carc.	0.5	8	Y	N	-	
**N.7**	78	7	adeno carc.	0.7	1	Y	Y,carc.	-	
**N.8**	118	5	adenosquamous carc.	0.5	10	Y	Y	-	liver
**N.9**	91	5	adenosquamous carc.	0.2	2	Y	Y,carc.	-	
**N.10**	63	5	adeno carc.	1.0	6	Y	N	-	
**N.11**	102	5	spindle cell	0.8	5	Y	N	+	
**N.12**	61	5	neuroendocrine carcinoid	1.0	0	Y	Y	nd	lung
**N.13**	105	4	adenosquamous carc.	0.4	0	N	N	nd	
									
**T16**									
**N.1**	90	6	adenosquamous carc. (IHC-)	1.0	4	Y	N	nd	
**N.2**	120	4	adenosquamous carc. (IHC-)	0.5	5	Y	N	-	
**N.3**	60	2	part adeno carc. + part spindle cell (IHC-)	2.0	3	Y	Y	-	lung (?)
**N.4**	113	5	adenosquamous carc. (IHC-)	0.8	3	N	N	-	
**N.5**	109	5	adenosquamous carc.	0.3	4	N	Y	-	
**N.6**	108	5	cribriform + adenosquamous carc.(IHC-)	1.0	7	Y	N	+	
									
**MP1**									
**N.1**	97	5	2 adenosquamous carc.	0.5	7	Y	N	-	
			1 papillair carc.	0.5					
**N.2**	100	6	papillair carc.	0.4	0	Y	Y	-	
**N.3**	53	7	endocrine carcinoid	2.0	0	N	N	-	
**N.4**	100	4	adenosquamous carc.	0.3	3	N	N	-	
**N.5**	98	3	adenosquamous carc.	2.0	0	Y	Y	-	lung
**N.6**	101	5	cribriform	2.0	2	N	Y	-	
									
T16*MP									
**N.1**	69	2	adeno carc. (IHC-)	1.5	2	N	N	-	
**N.2**	117	7	adenosquamous carc. (IHC-)	0.2	3	Y	Y	-	
**N.3**	95	3	adenosquamous carc. (IHC-)	0.2	4	Y	N	-	
**N.4**	101	4	papillair carc.	0.8	0	Y	Y,carc	-	
**N.5**	93	5	adenosquamous carc. (IHC-)	0.2	3	Y	Y,carc	-	
**N.6**	95	4	(adeno)squamous carc. (IHC-)	1.0	4	N	N	-	
**N.7**	55	5	(adeno)squamous carc.	1.0	0	Y	N	-	
